# Dimensions of Creativity in Secondary School High-Ability Students

**DOI:** 10.3390/ejihpe11030070

**Published:** 2021-08-24

**Authors:** Núria Arís Redó, María Ángeles Millán Gutiérrez, José-Diego Vargas Cano

**Affiliations:** Facultad de Educación, Universidad Internacional de la Rioja, 26006 Logroño, Spain; angelinesmillan@gmail.com (M.Á.M.G.); jose.vargas@unir.net (J.-D.V.C.)

**Keywords:** creativity, high ability, scientific creativity, secondary school students

## Abstract

The objective of this study was to analyze the dimensions of creativity in high-ability teenage students. Firstly, we reviewed the most relevant scientific contributions on creativity. Next, the dimensions of creativity in secondary school students who were previously identified as high-ability students were analyzed. The sample was obtained from 215 students, of which 31 were identified as high-ability students. The abilities associated with divergent thinking were assessed using the Torrance Test of Creative Thinking The fluency, flexibility, and originality dimensions were assessed with the Scientific-Creative Thinking Test. This study was conducted using a quantitative approach. Tests were administered during school hours from March to June 2019. They were corrected considering the protocols established by the original authors themselves. Data were analyzed using SPSS, version 24.0. The results provide evidence that high-ability students achieve higher scores in both the figurative-creativity and scientific-creativity dimensions. A significant relationship between creativity and high ability was therefore established. Students with high abilities and qualities require the educational support necessary to develop their talent. This study was of an exploratory nature and the results obtained contribute to developing future studies applying its findings in teenagers’ teaching–learning process.

## 1. Introduction

The different and most relevant scientific definitions and contributions related to creativity should be considered. In the early twentieth century, Wallas presented a description of the creative process by detailing four phases: preparation, incubation, illumination, and verification [1,2]. In the 1950s, Guilford researched the nature and measurement of creative thinking capacities [3]. In the 1960s, Rhodes defined four aspects that influence creativity, known as the four Ps (person, product, process, and press), emphasizing the interrelation among person, process, product, and environment for creative production [4]. The topic of creativity has resulted in different conceptualizations of approaches to and perspectives about this construct.

A first definition in this area was provided by Torrance in 1965, who considered creativity as the process of identifying problems or data gaps, forming ideas from hypotheses, testing and modifying these hypotheses, and communicating the results [5]. Torrance considered creativity a general ability that is implemented in different domains. In other words, the creative person has general abilities that can be implemented in all areas. This implies a definition of creativity as the capacity for formulating, verifying, and generating new ideas; assessing alternatives; looking for solutions; etc. Additionally, a number of dimensions of creativity were identified, comprising fluency regarding the number of answers [6]; flexibility, understood as the capacity to change the track of thought with regard to a particular task; originality, referring to the answers of new and innovative nature; and elaboration, referring to all the additional details. Based on all these dimensions, this author constructed an instrument that evaluates the capacity of people to produce different, original, and alternative ideas as a response to specific problems. By doing so, the creative potential can be assessed, considering both the qualitative and quantitative dimensions of divergent thinking. There is agreement that creativity is characterized by particular dimensions such as fluency, figurative reasoning, divergent thinking problem solving, and flexibility. Likewise, Renzulli established a relationship between high abilities and creativity. He considered high-ability people as being more proactive in the search for new solutions and therefore being more creativity-oriented in their own processes with the aim of achieving a different, new, and original result. This idea is closely linked with their claim that the creative capacity can arise under different circumstances [7]. Thus, Renzulli considered both creativity and the self-motivation task as behaviors related to high abilities. Similarly, Sternberg considered creativity to be an ability that must be developed and practiced [8]. Kaufman and Beghetto developed a four-category creativity model to help unveil the different nuances among the levels and types of creativity [9]. This model classifies a person according to the creativity they have in every facet of life. A given person could fit into multiple areas.

More creative students tend to be more intelligent, adventurous, extroverted, and self-confident. They also have a less favorable attitude toward school [10,11]. Other studies showed that high creativity scores in both the figural and verbal areas were associated with both high-test scores. To a lesser extent, high creativity scores were associated with high exam marks in mathematics and art. High creativity was associated with high verbal and quantitative IQ scores. Creativity increased with increasing age. The results suggested that although high levels of creativity may be associated with high levels of academic performance, this role is not causative [12].

Hu and Adey considered that creative thinking refers to the capacity to deal with problems in an original way. This implies a number of cognitive processes, such as problem identification, analysis, search for hypotheses, reformulation, interpretation of results, experimentation, etc., from an integrated vision [13]. They consider creative imagination as a process whereby original and innovative solutions to problems are generated. They based this consideration on the use of previous experiences and previous knowledge as a starting point to verify hypotheses and generate new solutions. They established that creative thinking is related to scientific thinking, since both pursue the search for new concepts, as well as raise new questions that allow the issues posed to be solved. It is understood that scientific thinking implies the cognitive processes applied to the specific actions of science, such as the generation of hypotheses, the use of causal reasoning, problem solving, etc. Scientific creativity is explained as the search for solutions to scientific problems. Through these cognitive processes, scientists pursue the achievement of an original result worth scientific recognition [13]. This model is the origin of the Scientific-Creative Thinking Test, which allows the research of scientific creativity. 

In 1965, Edwards and Tyler administered two creativity tests from the Torrance battery to 181 ninth grade students along with the School and College Achievement Test (SCAT) and Sequential Tests of Educational Progress (STEP) batteries [14]. A high-SCAT group consisting of Ss scoring in the upper third on SCAT but not on creativity was compared with a high-creativity group consisting of Ss scoring in the upper third on creativity but not on SCAT. The high-SCAT group was superior in terms of both school grade-point average and STEP scores. To test Torrance’s threshold hypothesis, a twice-talented group, high on both SCAT and creativity, was compared with the high-SCAT group. These two groups did not differ in STEP scores, but the grade-point average of the twice-talented group was significantly lower than that of the high-SCAT group [14].

The results previously obtained in the exploration of scientific creativity suggest that high-ability students are more fluent than other students, meaning they generate a greater number of ideas for the problems or questions posed. Ruiz Melero found that higher-intelligence students also showed better performance in most areas of the scientific creativity and dimensions test (fluency, flexibility, and originality). Previously, some authors reported a significant and positive relationship between intelligence and scientific creativity [15]. Along the same lines, the data showed that scientific creativity complements intelligence, academic performance, and socioeconomic status [4].

Lastly, Sternberg provided remarkable contributions about the triarchic theory of intelligence. Three types of intelligence were established: analytical, creative, and practical. Sternberg defined intelligence as a mental activity aimed at the intentional adaptation, selection, or transformation of real-world environments relevant to life [8]. Sternberg considered the relevance for context adaptation to transform it, taking circumstances into account. In coherence, they considered creativity an ability that can and must be developed [4,8].

As such, the aim of this study was to examine how to develop, enhance, and nurture creativity. As stated by Perry, creativity is a type of learning process where the teacher and pupil are within the same person [16].

We think that insufficient attention has been paid to high-ability students. Therefore, these secondary school students do not receive corresponding educational support. In this sense, the following statement should be highlighted: “the need for special attention becomes more obvious when it is verified that different children who receive the same educational treatment, they do not always obtain the same results” [17]. Students with high abilities and qualities require the educational support necessary to develop their talent. This study aims to make a first approach to this task [18].

Pfeiffer studied creative high-ability students. The characteristics they show are high fluency of thought and a large number of ideas to solve problems in original and new ways. Students who demonstrate these qualities and high abilities deserve the educational support to develop their talent. We aimed to make a first approach to this task [19]. We examined creativity in relation to high-ability students in compulsory secondary school. This study is exploratory in nature in that educational center. It was conducted in adolescents aged between 12 and 16 years, given the age of education in Spain. Based on this situation, we intend to apply our study to a larger sample in the near future.

### Objectives

The first of the research objectives was to analyze the creativity dimensions of high-ability students regarding fluency, flexibility, originality, and elaboration to determine if there are differences in these dimensions between high- and non-high-ability students.

From a research point of view, we aimed to observe not only if there are differences in creativity between high- and non-high-ability students, but in what specific dimensions of creativity such differences could be detected (fluency, flexibility, originality, or elaboration), evaluating these variables using a test free of cultural influence such as Torrance’s Test of Creative Thinking Model (TTCT) [20].

The second of the research objectives was to analyze the dimensions of scientific creativity to determine if there are differences in these dimensions between high-ability and non-high-ability teenage students. 

Similar to the previous objective, this test first allowed us to identify any differences in any of these dimensions (fluency, flexibility, and originality) between high-ability and non-high-ability teenage students, in this case, in a specific domain of competencies, the scientific dimension, which occupies a large space in the curricula of current educational systems (Scientific-Creative Thinking Test model (TPCC) [9]).

## 2. Materials and Methods

This study’s approach was quantitative in nature. This approach implies a cross-sectional study, which allows describing the study object at a precise moment without appreciating its evolution or antecedents.

### 2.1. Participants

The sample was obtained from a compulsory secondary school in a Mediterranean city of 500,000 inhabitants in Spain. Students age range was between 12 and 16 years old with 50.3% boys and 49.7% girls. This age range (12–16 years) corresponds to the compulsory secondary level education in this country. At the start of the study, of the 215 students, 31 were identified as high-ability students; the rest did not qualify for this category. In the application of the following tests, a difference can be observed in the number of students, due to their being carried out during school hours on an established day and time. Due to several problems, some of the 215 students were absent; therefore, the data reflect a smaller number of students.

### 2.2. Instruments

#### 2.2.1. Instruments Used to Collect Information

High-ability students were identified using two procedures: that proposed by Professors Castelló and Batlle and screening scales aimed at the detection of high creativity by Renzulli [21,22], which is a test that measures the student’s self-perception regarding learning, motivation, and creativity. Castelló and Batlle contributed to the research on high abilities by developing a protocol for the identification of high-ability students based on instruments of intellectual aptitudes and creativity [20]. This Castello and Batlle model emphasizes the existence of domains in which subjects can externalize greater skills. It is a study model widely used in our country due to its formulation [21]. Then, the Torrance Test of Creative Thinking was used (TTCT) [20] and, finally, the Hu and Adey Creative Scientific Thinking Test (TPCC, [9], which measure figurative and scientific creativity, respectively.

These instruments were chosen because they best fit the objectives of this research and allow the evaluation of creativity adjusted to the sociocultural context of the analyzed sample, so that greater objectivity and reliability could be achieved in the evaluation and subsequent statistical analysis. They were also known to evaluators and are easy to administer in an educational center context such as that used in this research. These two instruments were used, which are detailed below.

#### 2.2.2. Torrance Test of Creative Thinking (TTCT)

The Torrance Test of Creative Thinking was designed as an instrument to measure divergent thinking abilities. The test comprises three games where the student is asked to design a drawing, to elaborate a story, and to trace several parallel lines from which to draw the maximum number of possible designs. The reliability obtained by the author was 0.50 [20]. However, in later studies, the inter-judge reliability index was higher (90) [8]. In a later study [23], a reliability of 0.77 was reported. In the present study a reliability of 0.78 was obtained, which is considered acceptable. In the research, the test obtained an adequate inter-judge reliability, with Pearson’s correlation coefficients between 723 and 882.

We were interested in analyzing creativity in its different components, not considered as a unique ability, through the evaluation through the Torrance Test of Creative Thinking Model [20], which allows differentiating scores into fluency, flexibility, originality, and elaboration.

#### 2.2.3. Scientific-Creative Thinking Test

Based on the Torrance Test, the Scientific-Creative Thinking Test is an instrument used to assess scientific creativity that was constructed by Hu and Adey; it measures the fluency, flexibility, and originality dimensions. Students are requested to detail all the scientific uses they would assign to a piece of glass. Regarding this request, they must ask scientific questions and provide answers to others. The authors considered all areas grouped together in a general factor of scientific creativity. In the research carried out by the authors, the test obtained a satisfactory reliability index (α = 0.893) and an adequate interjudge reliability, with Pearson’s correlation coefficients between 0.793 and 0.913. With reference to the validity of the study, the factor analysis by Hu and Adey indicated that all items converged in a single factor that explained 63% of the variance [13].

Contrasting the results of both TTTC [20] and TPCC [9] tests, we compared the results to detect possible differences between both tests and analyze which test would be more sensitive for detecting the different nuances in the creativity of high-ability students.

#### 2.2.4. Procedures and Data Analysis

Tests were administered in the educational center during school hours from March to June 2019. They were corrected considering the protocols established by the original authors themselves. Then, the obtained data were treated using SPSS, version 24.0.

In the descriptive analysis of the variables, the minimum and maximum values, average, standard deviation, and statistical frequency were determined. Asymmetric rates and kurtosis were used to analyze the normality of the study variables. Likewise, the Kolmogorov–Smirnov test was used. Statistical tests were used to analyze the average difference between groups: parametric (Student’s t-test and ANOVA) or non-parametric (chi-square and Mann–Whitney U-test) tests were used according to the nature of variables. The relationship between variables was studied using Pearson’s correlation coefficient and the chi-square test.

## 3. Results

First, descriptive statistics were obtained. Table 1 lists the minimum and maximum values, average, and standard deviations, as well asymmetry and kurtosis scores. These results could indicate that high-ability students actually demonstrated that they are capable of manifesting higher scores in the creativity dimensions evaluated.

**Figure 1:**
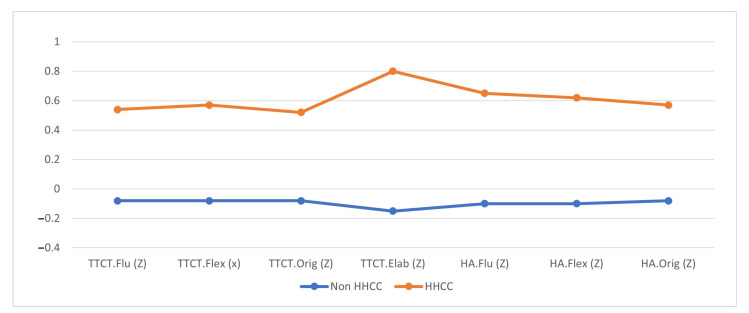
Scores in the different dimensions of creativity for both high-ability and non-high-ability students (standardized mean scores (Z) for creativity variables: non-HHCC, *n* = 177/178; HHCC, *n* = 31.

Secondly, to further elaborate upon the previous results, descriptive statistics were obtained for each student group (high-ability vs. non-high-ability). Figure 1 shows a graph of the average for both groups. From the graph, high-ability students obtained higher scores for all creativity dimensions, both in the Torrance Test (TTCT) and in the Scientific-Creativity Test by Hu and Adey [13,20].

To verify if these differences were statistically significant, relevant pertinent statistical tests were performed. In the case of TTCT [20] variables, the Mann–Whitney U-test was used, provided these variables were not distributed in a standard form, as analyzed in Table 1. The test indicated significant differences (*p* < 0.05) for the four TTCT dimensions, always in favor of high-ability students (Table 2). Once again, creativity was related to greater fluency, flexibility, originality, and elaboration in high-ability students.

In the case of dimensions measured by the TPCC test that assess scientific creativity, Student’s t-test for independent samples was used, since variables were distributed in a standard form. The tests results showed that differences were statistically significant regarding the dimensions of fluency, flexibility, and originality, always in favor of the high-ability students (Table 3).

## 4. Discussion

Through this study, we aimed to better understand the high abilities of secondary school students in the age range between 12 and 16 years old. The study was also conducted to deeply investigate the relationship between creativity and high ability, as well as to identify how the creative dimensions appear in more able students. Both creativity and intellectual giftedness are complex constructs, and it is often difficult to delimit them. Therefore, their assessment is expected to be complex.

The dimensions of creativity connected to high-ability students were intended to be researched from an exploratory perspective in this study. More precisely, it was relevant to obtain a deeper understanding of the behavior of figurative creativity (as measured by TTCT and by TPCC in high-ability students). The results of this study indicate high-ability students obtain higher scores in terms of fluency, flexibility, originality, and elaboration (figurative creative (TTCT) and dimensions (fluency, flexibility, and originality) of scientific-creativity (TPCC) [13,20]. Hence, we established a relationship between creativity and high abilities. A study on the validity of the Renzulli scales with Jordanian students was carried out by Hana and Ali, using the teacher’s perception scale [7]. To research the external validity of the scale, they compared the averages obtained by the students considered as high-abilities and non-high-abilities using other procedures (IQ). This research shows that high-abilities students (according to the IQ criterion) obtain higher scores than their fellow students on the Renzulli scale.

Belmonte-Lillo found no statistically significant differences between high-ability and divergent thinking (TTCT) but they were found in the elaboration dimension (in favor of cognitive high-ability groups [23]. In our preliminary study, significant differences were observed in terms of intelligence, in favor of cognitive high-ability groups. The data showed that high abilities are linked to greater possibilities of improving, perfecting, or adding new elements to an initial idea. This is the capacity of being creative and seems to be present in the high-ability students evaluated.

The results obtained in this exploration of scientific creativity agree with those of other studies, which suggests high-ability students have greater fluency than other students [24]. They generate a larger number of ideas in response to the problems or questions raised. Likewise, Ruiz Melero found that students with higher intelligence also performed better in most areas of the scientific creativity test and dimensions (fluency, flexibility, and originality) [15]. Previously, other authors reported a positive and significant relationship between intelligence and scientific creativity [16]. Similarly, other findings showed the scientific creativity complements intelligence, academic performance, and socioeconomic status [25,26].

It is necessary to place these results in context, as the authors of the Scientific-Creativity Test (TPCC) compared students according to how skillful they were in the sciences. Significant differences were found (*p* ≤ 0.01) between low- and average-ability students, but no differences were found between average- and high-ability students according to the instrument′s own scales, though the scores in scientific creativity by high-ability students were higher than those obtained by average-ability students. The authors concluded that creativity is a necessary condition for sciences, but not enough for the expression of the scientific creativity of secondary school students [20,27]. Our team also considers the challenges of evaluating these adolescents, which consist of using evaluation scales; questionnaires for parents, teachers, and the children themselves; and accurately collecting all the information from the context of the teaching and learning environment.

## 5. Conclusions

It will be important to have more instruments that consider cognitive abilities, creative abilities (of a general and specific nature), performance in the teaching–learning process, the potential to achieve excellence, as well as the motivational aspects and the context where that potential is developed. The results obtained in this study indicate that high-ability students obtain higher scores for the figurative creativity and scientific-creativity dimensions (fluency, flexibility, and originality). All this allows the establishment of a significant relationship between creativity and high-ability students.

These results agree with those of previous studies where high-ability students contributed a larger number of new ideas to a question or problem raised [15,23].

The results obtained coincide with other studies in the idea that it is appropriate to combine several methods, test, and trials to evaluate high abilities and creativity as complementary aspects [26,28]. They are complex and difficult-to-identify constructs. The results obtained in our study also correspond, with slight deviations, to the real proportions in Castelló and Batlle [21]. Regarding to our sample, 31 of 215 students were identified, which represents 14%. This percentage aligns with the findings of other authors such as Renzulli, who constructed comprehensive identification models [22,28]. However, it contrasts with more restrictive models, which estimated that only 5% of the population has high abilities [17,29].

From the point of view of psychoeducational intervention, we propose taking advantage of the creative potential of high-ability students to improve the teaching–learning activities by enriching the focus on the dimensions of fluency, flexibility, and originality; the learning based on problems and by project; or cooperative learning. The contributions of this study in the teaching and learning process of high-ability students are clear [30,31], and they coincide with the contributions of the Gagne model. This model helps us to process the information and to understand the information being processed or transformed as structures are elaborated and reworked [32]. Therefore, the largest number of high-ability students should be identified to be able to offer the attention required to enhance their talent and abilities [17,33]. This study confirms that it is necessary to identify more students in order to better able to serve them [26,34]. The final aim will be to apply the findings to the teaching–learning process [35].

We point out future lines of research as follows:

- Contrasting these results with a larger research sample. In the context of preliminary research of our study, it has been found that with the methodology of this research it is possible to improve the detection of high-ability students and their levels of creativity. Unfortunately, this is not a procedure that is widespread in our educational system.
- Contrasting these results with those of students of other ages (younger boys and girls, young adults, etc.) to determine the evolutionary dimensions of creativity and high abilities.
- Designing educational proposals that allow the improvement in the educational attention to high-ability and high-creativity students. Our intention is to extend this detection methodology so that it is incorporated into the orientation teams of the educational centers. In future research, in collaboration with the educational administration, training activities for counselling teams are planned to improve their diagnostic skills and offer strategies to improve educational intervention.

## 6. Limitations

Finally, as this study was exploratory in nature, the results obtained will help us to develop further studies that will contribute to delimiting the relationships between creativity and high abilities. We are aware that one of the limitations of our study is the need to develop the actions required for educational implementation. We intend to continue this research in order to develop these educational actions.

Other limitations are related to the size of the sample, which was small but sufficient considering this was a first approach to the subject. Regarding the experience of this first study, we intend to continue research with a larger sample. The age of the sample, centered on the beginning of adolescence with variable school attendance, resulted in a limitation on the ability to deliver the tests to the students.

Finally, another limitation is the difficulty of defining the concepts of high ability and creativity. Complexities are inherent in the relationship between high ability and creativity, and defining their complementarity motivates us to conduct a systematic review in the near future. 

## Figures and Tables

**Table 1:** Descriptive statistics for creativity dimensions measured for Torrance TTCT test [22] and TPCC test by Hu and Adey [13].

	N	Min.	Max.	Average	SD	Asym.	Kurtosis
TTCT Fluency	211	1	40	21.21	8.20	0.11	−0.26
TTCT Flexibility	211	1	40	19.88	8.12	0.21	−0.17
TTCT Originality	211	1	87	33.74	16.34	0.41	−0.09
TTCT Elaboration	211	4	126	38.30	19.70	0.94	1.54
HA Fluency	212	10	90	35.50	13.67	0.86	0.87
HA Flexibility	212	5	49	21.19	8.05	0.69	0.60
HA Originality	212	8	139	39.93	20.18	1.17	2.49

**Table 2:** Comparison of averages for Torrance Test of Creative Thinking [23].

	Non-HHCC (*n* = 177)			HHCC (*n* = 31)			Comparison Averages	
	Average	SD	Rank	Average	SD	Rank	U ^1^	*p* ^2^
TTCT Fluency	20.64	7.61	4.76	25.58	9.81	3.90	1981	0.014
TTCT Flexibility	19.27	7.51	4.80	24.42	9.82	3.94	1979	0.013
TTCT Originality	32.69	15.30	4.96	41.94	19.13	4.90	1991	0.015
TTCT Elaboration	35.76	17.70	4.47	53.81	23.56	5.53	1441.5	<0.001

^1^ Mann–Whitney U-Test; ^2^ asymptotic signification (bilateral).

**Table 3:** Comparison of averages for Hu and Adey’s test [8].

	Non HHCC (*n* = 178)			HHCC (*n* = 31)			Student’s *t*-Test
	Average	SD	Rank	Average	SD	Rank	
HA Fluency	34.16	13.20	80	44.48	13.13	49	*t* (207) = −4.021; *p* < 0.001
HA Flexibility	20.43	7.56	44	26.26	9.13	37	*t* (207) = −3.839; *p* < 0.001
HA Originality	38.41	19.47	131	50.39	21.63	89	*t* (207) = −3.108; *p* = 0.002

## Data Availability

The data presented in this study are available on request from the authors.
